# WBC image classification and generative models based on convolutional neural network

**DOI:** 10.1186/s12880-022-00818-1

**Published:** 2022-05-20

**Authors:** Changhun Jung, Mohammed Abuhamad, David Mohaisen, Kyungja Han, DaeHun Nyang

**Affiliations:** 1 Department of Cyber Security, Ewha Womans University, 52, Ewhayeodae-gil, Seodaemun-gu, Seoul, 03760 Republic of Korea; 2 Department of Computer Science, Loyola University Chicago, 1032 W Sheridan Rd, Chicago, 60660 USA; 3 Department of Computer Science, University of Central Florida, 4000 Central Florida Blvd, Orlando, FL 32816 USA; 4 Department of Laboratory Medicine and College of Medicine, The Catholic University of Korea Seoul St. Mary’s Hospital, 222, Banpo-daero, Seocho-gu, Seoul, 06591 Republic of Korea

**Keywords:** White blood cell, Classification, Medical image, CNN, Deep learning

## Abstract

**Background:**

Computer-aided methods for analyzing white blood cells (WBC) are popular due to the complexity of the manual alternatives. Recent works have shown highly accurate segmentation and detection of white blood cells from microscopic blood images. However, the classification of the observed cells is still a challenge, in part due to the distribution of the five types that affect the condition of the immune system.

**Methods:**

(i) This work proposes W-Net, a CNN-based method for WBC classification. We evaluate W-Net on a real-world large-scale dataset that includes 6562 real images of the five WBC types. (ii) For further benefits, we generate synthetic WBC images using Generative Adversarial Network to be used for education and research purposes through sharing.

**Results:**

(i) W-Net achieves an average accuracy of 97%. In comparison to state-of-the-art methods in the field of WBC classification, we show that W-Net outperforms other CNN- and RNN-based model architectures. Moreover, we show the benefits of using pre-trained W-Net in a transfer learning context when fine-tuned to specific task or accommodating another dataset. (ii) The synthetic WBC images are confirmed by experiments and a domain expert to have a high degree of similarity to the original images. The pre-trained W-Net and the generated WBC dataset are available for the community to facilitate reproducibility and follow up research work.

**Conclusion:**

This work proposed W-Net, a CNN-based architecture with a small number of layers, to accurately classify the five WBC types. We evaluated W-Net on a real-world large-scale dataset and addressed several challenges such as the transfer learning property and the class imbalance. W-Net achieved an average classification accuracy of 97%. We synthesized a dataset of new WBC image samples using DCGAN, which we released to the public for education and research purposes.

## Background

White blood cells (WBCs) are one type of blood cells, besides red blood cell and platelet, and are responsible for the immune system, defending against foreign substances and bacteria. WBCs are typically categorized into five major types: neutrophils, eosinophils, basophils, lymphocytes and monocytes. Neutrophils consist of two functionally unequal subgroups: neutrophil-killers and neutrophil-cagers, and they defend against bacterial or fungal infections [2]. The number of eosinophils increase in response to allergies, parasitic infections, collagen diseases, and disease of the spleen and central nervous system [3]. Basophils are mainly responsible for allergic and antigen response by releasing chemical histamine causing the dilation of blood vessels [4]. Lymphocytes help immune cells to combine with other foreign invasive organisms such as microorganisms and antigens, in order to remove them out of the body [5]. Monocytes phagocytose foreign substances in the tissues [6]. The usual distribution of these five classes is 62%, 2.3%, 0.4%, 30% and 5.3% among WBCs in the body [7]. This distribution of WBC describes the condition of the immune system. Considering the complexity of manually estimating the distribution of WBC, *e.g.,* by consulting a human expert, many studies have introduced methods for automating the process through WBC segmentation, detection, and classification. Despite these numerous studies, which are greatly focused on the segmentation and detection tasks, less attention has been given to the WBC classification task and factors impacting the accuracy and performance of the task.

Accurate WBC classification is also beneficial for diagnosing leukemia, a type of blood cancer in which abnormal WBCs in the blood rapidly proliferate, decreasing the number of normal blood cells making the immune system vulnerable to infections In the US, around 60,000 people are diagnosed with leukemia every year, and around 20,000 people die of leukemia annually. From 2011 to 2015, leukemia was the sixth most common cause of cancer-caused death in the US [8]. There are various types of leukemia, including ALL (Acute lymphocytic leukemia), AML (Acute myelogenous leukemia), CLL (Chronic lymphocytic leukemia), CML (Chronic myelogenous leukemia). Chronic leukemia progresses more slowly than acute leukemia which requires immediate medical care. Acute leukemia is characterized by proliferation of blasts, CLL is characterized by increased lymphocytes while CML shows markedly increased neutrophils and some basophils in the blood [9]. Therefore, accurate classification of WBCs contributes to the diagnosis of leukemia.

Recent advancements in the field of computer vision and computer-aided diagnosis show a promising direction for the applicability of deep learning-based technologies to assist accurate classification and counting of WBC. Convolutional neural network (CNN) is one of the most common and successful deep learning architectures that have been utilized for analyzing and classifying medical imagery data [10–13]. In this paper, we propose W-Net, a CNN-based network for WBC images classification. W-Net consists of three convolutional layers and two fully-connected layers, and they are responsible for extracting and learning features from WBC images and classifying them into five classes using a softmax classifier. In comparison to state-of-the-art methods, W-Net shows outstanding results in terms of accuracy. Further, we investigate the performance of several deep learning architectures in performing the WBC classification task. We applied and compared the performance of several architectures including W-Net, AlexNet [14], VGGNet [15], ResNet [16], and Recurrent Neural Network (RNN). Moreover, we compared the utilization of different classifiers such as softmax classifier and Support Vector Machine (SVM) on top of the adopted models. Moreover, we explore the effects of pre-training W-Net using public datasets, such as the LISC public [17], on its performance. Understanding the importance of large-scale datasets on the models’ performance, we generate new WBC images using GAN [18] to augment current educational and research datasets.

### Contributions

The contributions of this paper are as follows. 1 We propose ❶ W-Net, a CNN-based network, designed to accurately classify WBCs while maintaining a high efficiency through minimal depth of the CNN architecture. ❷ We evaluate the performance of W-Net using a real-world large-scale dataset that consist of 6562 real images. ❸ We address and handle the problem of imbalanced classes of WBCs and achieve an average classification accuracy of 97% for all classes. ❹ We show how W-Net which consists of three convolutional layers stands among most popular CNN-based architectures, in the field of image classification and computer vision, in performing the WBCs classification task. ❺ Serving the purpose of advancing the task, we studied the applicability of transfer learning and generating larger datasets of WBC images using GAN for the public release. ❻ We generate and publicize synthetic WBC images using Generative Adversarial Network to be used for education and research purposes. The synthetic WBC images are verified by experiments and a domain expert to have a high degree of similarity to the original images. The pre-trained W-Net and the generated WBC dataset are available for the public.

### Organization

The rest of the paper is organized as follows: in “Related works” section, we review literature. We introduce our model W-Net in “Methods” section. We evaluate W-Net through various experiments on WBC images in “Experiments” section. Our design choices and the experiment result are discussed in “Design considerations for W-Net” section. We release a new WBC dataset using GAN in “Dataset sharing” section. Finally, we conclude our study in “Conclusion” section.

## Related works

### Previous works

Analysis of white blood cells (WBC) has vital importance in diagnosing diseases. Distribution of the five WBC types, (basophils, eosinophils, lymphocytes, monocytes and neutrophils) reflects highly on the condition of the immune system. Analyzing the components of WBCs requires performing segmentation and classification processes. The traditional analysis of WBC includes observing a blood smear on a microscope and using the visible properties, such as shapes and colors, to classifing the blood cells. However, the accuracy of the WBCs analysis depends significantly on the knowledge and experience of the medical operator [19]. This makes the process of analyzing of WBCs using conventional methods time-consuming and labor-intensive [19–21]. Therefore, many studies have proposed computer-aided technologies to facilitate the WBC analysis through accurate cell detection and segmentation to reduce the manual efforts needed by human experts. For instance, Shitong and Min [22] have proposed an algorithm based on fuzzy cellular neural networks to detect WBCs in microscopic blood images as the first key step for automatic WBC recognition. Using mathematical morphology and fuzzy cellular neural networks, the authors achieved a detection accuracy of 99%. The detection of WBCs is followed by a segmentation process, which segments the image into nucleus and cytoplasm regions. This task has been pursued by several studies providing accurate segmentation using a variety of methods. The most common approach for nuclei segmentation is the clustering based on extracted features from pixels values [23, 24]. The literature shows a successful nuclei segmentation using different clustering techniques, such as K-means [25], fuzzy K-means [24], C-means [24], and GK-means [26]. Among other unsupervised techniques for nuclei segmentation beside clustering, many studies utilized thresholding [21, 27–31], arithmetical operations [32], edge-based detection [24, 31], region-based detection [31], genetic algorithm [33], watershed algorithm [31], and Gram-Schmidt orthogonalization [17].

The literature on WBC segmentation process is very rich and provides valuable insights for the WBC identification. Andrade et al. [23] provides a survey and a comparative study on the performance of 15 segmentation methods using five public WBC databases. Some of these works are dedicated to the separation of adjacent cells, while many others addressed particularly the separation of overlapping cells. After the segmentation process, the WBC image classification or identification process is conducted. The distinction between the task of WBC identification and WBC image classification is the identification process aims to detect and identify leucocytes in an image, while the classification process aims to distinguish the different types of WBC. Even though many studies are dedicated to segmentation and identification task, fewer researches are addressed the classification of the WBCs. The literature shows that classification methods used for this purpose include the K-Nearest Neighbor (KNN) classifier [20, 28], Bayesian classifier [21, 28, 34], SVM classifier [17, 19, 26, 28, 35], Linear Discriminant Analysis (LDA) [36], decision trees and random forest classifier [28, 37], and deep learning [17, 27, 32, 35, 38, 39].

Recently, deep learning-based methods have been utilized for WBC classification and segmentation tasks [40–42]. Patil et al. [40] incorporated canonical correlation analysis with CNN to extract and train on multiple nuclei patches with overlapping nuclei for WBC classification. Toğaçar et al. [41] have utilized multiple CNN-based models, namely, AlexNet, GoogLeNet, and ResNet-50, for feature extraction and adopted quadratic discriminant analysis for classifying WBCs. Their method achieved an accuracy of 97.95% on a dataset of four categories: Neutrophil, Eosinophil, Monocyte, and Lymphocyte. Mohamed et al. [43] have investigated the use of deep CNN models over different shallow classifiers for WBC classification. For example, using a logistic regression classifier, extracting features using MobileNet-22 enabled a classification accuracy of 97.03%. Banik et al. [44] explored the use of combining features from different layers of CNN model to classify WBC in the BCCD dataset. Karthikeyan et al. [45] proposed the LSM-TIDC approach to classify WBCs in blood smear images where a multi-directional model is used to extract texture and geometrical features that are then fed to a CNN model. Kutlu et al. [46] proposed using Regional-Based CNN model for WBC classification in blood smear images. Many other approaches have been proposed to tackle various challenges in the field of WBC using traditional machine learning and deep learning-based methods. Khan et al. [42] provided a comprehensive review of such practices and their impact on the field. Table 1 shows an overview of the performance and methods of the related works.

**Table 1:** Related work highlighting the used datasets, their size, number of classes (C), employed methods, and accuracy

Study	Dataset	Size	C	Methods	Performance
Wang et al. [19]	Private: hyperspectral blood cell images	N/A	5	Morphology, spectral analysis and SVM	90.00%
Dorini et al. [20]	CellAtlas	100	5	Morphological transform. and KNN	78.51%
Nazlibilek et al. [27]	Kanbilim dataset [73]	240	5	Thresholding, ANN and PCA	95.00%
Prinyakupt et al. [21]	Private dataset: Rangsit University and	PD: 555	5	Thresholding and NB	PD: 93.70%
	CellaVision dataset	CV: 2477			CV: 92.90%
Abdeldaim et al. [28]	ALL-IDB2	260	2	Thresholding, KNN, SVM, NB and DT	KNN: 96.01%
					SVM: 93.89%
					NB: 89.97%
					DT: 86.81%
Hegde et al. [32]	Private: Kolkata Municipal Corporation	117	5	Arithmetical operations and ANN	96.50%
Ghosh et al. [74]	ALL-IDB	260	2	CNN	97.22%
Rezatofighi et al. [17]	Private: Imam Khomeini Hospital	400	5	Gram-Schmidt, SVM and ANN	98.64%
Habibzadeh et al. [38]	Private [75]	352	4	CNN	93.17%
Liang et al. [76]	BCCD [77]	364	4	RNN (LSTM) and CNN	90.79%
Rawat et al. [39]	Private [78]	160	4	Ensemble ANN	95.00%
Ramesh et al. [36]	Private: University of Utah	320	5	LDA	93.90%
Putzu et al. [79]	ALL-IDB	260	2	SVM	92.00%
Mathur et al. [34]	Private	237	5	NB	92.72%
Ghosh et al. [37]	Private: Kolkata Municipal Corporation	150	5	Region-based segmentation	N/A
				Mathematical morphology	N/A
				Fuzzy logic and RF	N/A
Su et al. [35]	CellaVision [80]	450	5	Mathematical morphology	HCNN: 88.89%
				Hyperrectangular composite NN	SVM: 97.55%
				SVM and MLP	MLP: 99.1%
Patil et al. [40]	BCCD [77]	12,442	4	CNN and RNN	95.89%
Toğaçar et al. [41]	BCCD [77]	12,435	4	AlexNet, GoogLeNet and ResNet	97.95%
Mohamed et al. [43]	BCCD [77]	12,500	4	MobileNet-22	97.03%
Banik et al. [44]	BCCD, ALL-IDB2, JTSC, and CV [80]	13,371	4	CNN	94.00%
Karthikeyan et al. [45]	BCCD [77]	12,500	4	LSM-TIDC	N/A
Kutlu et al. [46]	BCCD [77] and LISC [17]	12,500	5	Regional-based CNN	97.52%
**W-Net (this work)**	**Private: The Catholic University of Korea**	**6562**	**5**	**CNN**	**97%**
**W-Net (this work)**	**LISC public data **[17]	**254**	**5**	**CNN and further training**	**96%**

The parts in bold mean our model

### CNN with medical images

Due to the vast success in a variety of applications, CNN has been adopted in several medical applications where imagery inputs are analyzed for diagnosis or classification. In the field of medical imaging, CNN has been successfully utilized for histological microscopic image [47], pediatric pneumonia [48], diabetic macular edema [48], ventricular arrhythmias [49], thyroid anomalies, mitotic nuclei estimation [50, 51], neuroanatomy [52], and others [10–13, 53–59]. Kermany et al. [48] showed that CNN can detect diabetic macular edema and age-related macular degeneration with high accuracy and with a comparable performance of human experts. The authors also demonstrated the applicability of CNN in diagnosing pediatric pneumonia from chest X-ray images. Alexander et al. [47] have provided the state-of-the-art performance (by the publication date) using CNN for histopathological image classification on the dataset of the ICIAR 2018 Grand Challenge on Breast Cancer Histology Images. Acharya [49] have shown that CNN can be used to accurately detect shockable and non-shockable life-threatening ventricular arrhythmias. Wachinger et al. [52] proposed DeepNAT, a CNN-based approach for automatic segmentation of NeuroAnaTomy in magnetic resonance images. The authors showed that their approach provided comparable results to those of state-of-the-art methods.

## Methods

This section provides a description of the dataset used in this study, the pre-processing steps for the WBC images, and the proposed CNN-based architecture for WBC classification. The dataset was provided by The Catholic University of Korea (The CUK), and approved by the Institutional Review Board (IRB) of The CUK [60]. The experimental protocols and informed consent were approved by the Institutional Review Board (IRB) of The CUK [60].

### Dataset

We use a real-world dataset of 6562 images that belong to five WBC types, namely, neutrophil, eosinophil, basophil, lymphocyte, and monocyte. The dataset was provided by The Catholic University of Korea (The CUK), and approved by the Institutional Review Board (IRB) of The CUK [60]. The images were captured by Sysmex DI-60 machine [61], and provided with 360 × 361 × 3 (3 channels, RGB) image size. Table 2 shows the number of images per class: 2006 neutrophils (NE) images, 1310 eosinophils (EO) images, 377 basophils (BA) images, 1676 lymphocytes (LY) images and 1193 monocytes (MO) images. The class distribution in our dataset is 30%, 20%, 6%, 26% and 18% for the five classes.

**Table 2:** The number of five type samples in the dataset

	NE	EO	BA	LY	MO
The # of Imgs.	2006	1310	377	1676	1193
Distribution	30%	20%	6%	26%	18%

### Pre-processing of WBC images

**Fig. 1:**
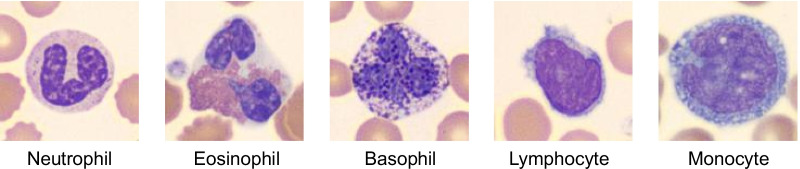
Neutrophil, eosinophil, basophil, lymphocyte and monocyte from the left. These were cropped and rescaled with 128 × 128 × 3 for efficient training

**Fig. 2:**
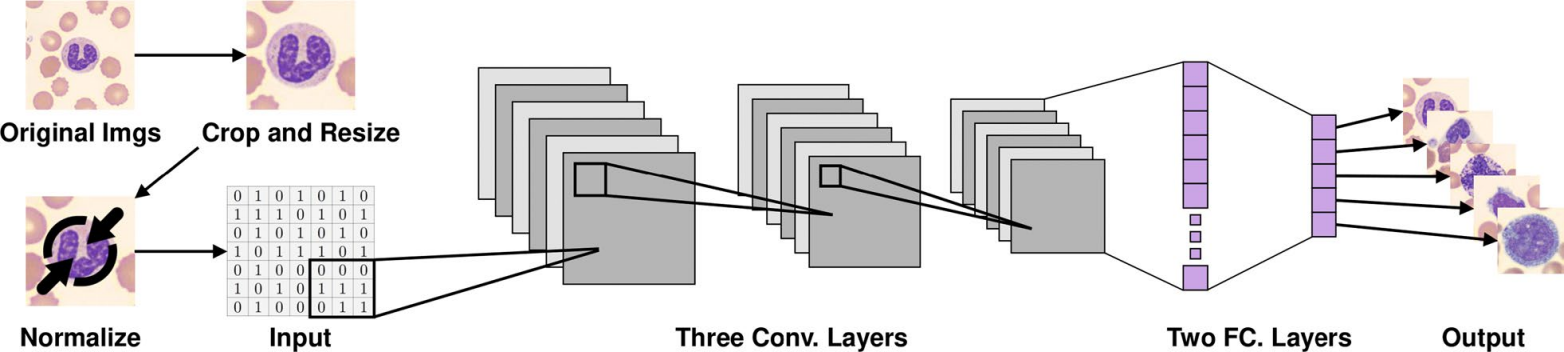
An overview of the pre-processing and the proposed CNN-based architecture for WBC image classification. The pre-processing consists of cropping, re-sizing and normalizing. Three convolutional layers (including three pooling layers) are in charge of extracting and learning features, and two fully connected layers are in charge of classification

Prior to the model creation and training, WBC images are pre-processed using three steps: ❶ image border cropping, ❷ image re-sizing, and ❸ image normalization. To eliminate the external borders of the image and to focus on the WBC, we remove the top 80 pixels, the bottom 81 pixels, the left 80 pixels, and the right 80 pixels of the image. The resulting cropped images, *i.e.,* images with a size of 200 × 200 × 3, are then re-sized to 128 × 128 × 3 for properly fitting them into a GPU memory and for efficient processing. Samples of the processed images are shown in Fig. 1. The image normalization process was applied to reduce the heterogeneity of the RGB distribution in the images and to prevent over/underflow. This step is shown in Fig. 2.

### W-Net: architecture and design

We introduce our CNN-based model architecture for WBC image classification. As illustrated in Fig. 2, W-Net consists of three convolutional layers and two fully-connected layers, and they are responsible for extracting and learning features from WBC images to accurately classifying them into five classes using a softmax classifier. Each convolutional layer has a kernel size of 3 × 3 with stride of size 1 and uses ReLU activation function *f*(*x*) = *max*(0, *x*). The first convolutional layer has 16 filters, the second has 32 filters and third has 64 filters. After each convolutional layer, there is a max-pooling layer of size 2 × 2 with stride of size 2 and zero padding. We also use dropout regularization with *p* = 0.6 [62] to prevent overfitting in each convolutional layer. The output of the third convolutional layer is flattened and fed into the first fully connected layer which has 1024 units. ReLU activation, and dropout with *p* = 0.6 are followed. The second fully connected layer has five units (five classes of WBC) and is followed by softmax classifier to map the output (features) to one of the five classes. The network has a total size of 16,806,949 trainable parameters. The model parameters were initialized using Xavier uniform initializer W ~ U[− x, x], where *x* = *sqrt*(6/(*in* + *out*)). The training of models is guided by minimizing the softmax-cross-entropy loss function $- \sum\nolimits_{x} {p(x)log q(x)}$, where $q(x_{i} ) = exp (x_{i} )/\sum\nolimits_{j} {exp (x_{j} ))}$ using Adam optimizer $\Delta \theta_{t} = - n \cdot \hat{m}_{t} /\sqrt {\hat{v}_{t} + \epsilon }$ [63] with a learning rate of 0.0001. The training process is conducted with different batch sizes and terminated by the conclusion of 500 training epochs. The evaluation of the models is conducted using a tenfold cross-validation approach [64]. The structure is illustrated in Table 3. The hyperparameters are described in Table 4. Design choices for W-Net are discussed in “Design considerations for W-Net” section.

**Table 3:** The structure of five layers (Conv. and FC.) for W-Net

Layers	Output size	Structure
1st Conv.	65,536	3 × 3 kernel, 1 stride, 16 filters
		2 × 2 max-pool, 2 strides, 0 pad
2nd Conv.	32,768	3 × 3 kernel, 1 stride, 32 filters
		2 × 2 max-pool, 2 strides, 0 pad
3rd Conv.	16,384	3 × 3 kernel, 1 stride, 64 filter
		2 × 2 max-pool, 2 strides, 0 pad
1st FC.	1024	1024 units
2nd FC.	5	5 units

**Table 4:** Hyperparameters for all the models

Architecture	Learning rate	Decay	Momentum	Dropout	Batch size	Epochs	Hidden unit
W-Net	0.0001			0.6	256	500	
W-Net with SVM	0.0001			0.6	256	500	
AlexNet	0.001	0.0005	0.9	0.5	128	90	
VGGNet	0.000001			0.5	1	300	
ResNet50	0.001	0.0001	0.9		32	50	
ResNet18	0.001	0.0001	0.9		32	50	
RNN	0.01				64		32

## Experiments

We show the performance of W-Net for WBC classification and compare the softmax classifier of W-Net with SVM. We show that W-Net provides remarkable results in the WBC classification by comparing it to the prior work. We also show how the number of layers affects performance. The comparison includes AlexNet, VGGNet, ResNet and RNN models. For transfer learning, we provide insights on adopting pre-trained W-Net to gain higher WBC classification performance on public datasets. ROC curve and AUC are a useful method for evaluating a system in medical area and are usually used to classify a binary task such as a diagnosis. However, we remark that our results are only based on an accuracy, because the output of our model is multiple-class not the binary.

### W-Net performance

**Fig. 3:**
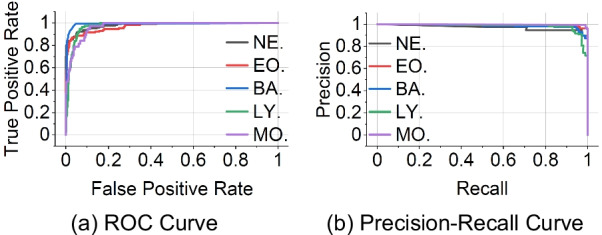
**a** Provides ROC curve of our W-Net model based on the idea of one versus rest for multi-class classification, and **b** shows Precision–Recall curve. In **a**, each class achieves an AUC of 0.97 on average and achieves an AUC of 0.98 on average in **b**

Table 10 in “Appendix 1” shows the accuracy achieved by W-Net using tenfold cross-validation approach. Conducting the experiments required 33.87 h of model’s training time. For the neutrophil, 1800 images were used for training and 206 images were used for testing in each fold, and the average accuracy was 98%. For the eosinophil, 1179 images were used for training and 131 images were used for testing in each fold, and the average accuracy was 97%. For the basophil, 340 images were used for training and 37 images were used for testing in each fold, and the average accuracy was 95%. For the lymphocyte, 1509 images were used for training and 167 images were used for testing in each fold, and the average accuracy was 97%. For the monocyte, 1074 images were used for training and 119 images were used for testing in each fold, and the average accuracy was 97%. The average overall accuracy of the five WBC classes was 97%. As shown in Fig. 3, it provides ROC curve and Precision-Recall (PR) curve in (a) and (b) respectively, based on the idea of one vs rest for multi-class classification. Each line in (a) represents each class of the five WBC classes, and our W-Net model achieved an AUC of 0.97 on average on ROC curve. On the one hand, it achieved an AUC of 0.98 on average on PR curve.

### W-Net versus W-Net-SVM performance

We compared softmax classifier of W-Net with SVM to demonstrate classifier’s abilities in performing the WBC classification task. We trained a W-Net model with SVM classifier (W-Net-SVM) using hinge loss function [65] *l*(*y*) = *max*(0, 1 −* t*ㆍ*y*) instead of softmax (W-Net). We followed the same experimental settings adopted in previous experiment including the training parameters, dataset, pre-processing steps, workstation environment, and tenfold cross-validation approach for the evaluation. The network has a total of 16,806,949 trainable parameters. Table 11 in “Appendix 1” shows the performance of W-Net-SVM using tenfold cross-validation in the WBC classification task. The training time of W-Net-SVM was 33.79 h. The achieved results for the neutrophil, eosinophil, basophil, lymphocyte, and monocyte classes are 98%, 97%, 87%, 98%, and 97%, respectively. The overall average accuracy of the five classes was 95%.

### WBC classification with AlexNet

This experiment adopts AlexNet architecture in the WBC classification task. AlexNet network consists of five convolutional layers and three fully-connected layers which apply ReLU activation function (in all layers except the last (softmax) layer). The training of AlexNet model is conducted by minimizing the softmax-cross-entropy loss function using the momentum optimizer $\theta_{t} = - \gamma \nu_{t - 1} - \eta g_{t}$ [66]. Using a cross-validation approach, the best training hyperparameters that achieved the best WBC classification accuracy are described in Table 4. We follow the same experimental settings adopted in previous experiments by using same dataset, pre-processing steps (except for the image size, we re-sized the images to 224 × 224 × 3 for AlexNet), workstation environment, and the tenfold cross-validation evaluation approach. The AlexNet-based network has a total of 46,767,493 trainable parameters. Table 12 in “Appendix 1” shows the performance of AlexNet using a tenfold cross-validation approach in the WBC classification task. The overall average accuracy is 84% (see Table 12 for details).

### WBC classification with VGGNet

We compared W-Net with VGGNet to demonstrate the effectiveness of W-Net in the WBC image classification. We trained a VGGNet-based model that consists of 16 convolutional layers and three full-connected layers, which followed with ReLU activation function. The model training is conducted using the minimization of the softmax-cross-entropy loss though Adam optimizer. Using a cross-validation method, the best training hyperparameters are described in Table 4. This experiment followed the same experimental settings adopted in previous experiments. The VGGNet-based model includes a total of 121,796,165 trainable parameters. The training of the VGGNet-based model required 510.59 h of training time. Table 13 in “Appendix 1” shows the results of the tenfold cross-validation of VGGNet-based model in the WBC classification. The overall average accuracy of the five classes is 44% (see Table 13 for details).

### WBC classification with ResNet

We adopt ResNet50 and ResNet18 networks for WBC classification, which consists of 50 and 18 convolutional layers, respectively. Both models are trained by minimizing the softmax-cross-entropy loss using momentum optimizer. Using a cross-validation approach, the best training hyperparameters to achieve the highest accuracy in the WBC classification task are described in Table 4. The training and evaluation of the models are in compliance with experimental settings adopted in previous experiments. The ResNet50 and ResNet18 models include a total of 23,544,837 and 14,722,931 trainable parameters, respectively. It required a training time of 8.38 h for ResNet50, and 3.51 h for ResNet18. Table 14 in “Appendix 1” shows the classification accuracy obtained by of ResNet50 model using the tenfold cross-validation approach, while Table 15 shows the results of ResNet18. The overall average accuracy of the five classes for ResNet50 is 51%. On the one hand, ResNet18 achieved the overall average accuracy of the five classes is 79% (see Tables 14 and 15 for details, respectively).

### WBC classification with RNN

We explore the capabilities of RNN in the WBC classification task. Using RNN for WBC image classification, we adopted the common approach by considering the image rows as sequences and the columns as timesteps. Since we the WBC images are 128 × 128 × 3 images, we feed the model with batches of 128 sequences of size 128 × 3. The RNN model adopted in this experiment consists of only one single hidden layer. The experimental settings for the training process are set with the following search space: learning rate = 0.0001, 0.001, 0.003, 0.01, 0.1, 0.3, batch size = 16, 32, 64, 128 and hidden units = 16, 32, 128, 256, 512, 1024. For hyper-parameter selection, we split 6562 images into train/test/validation sets by 5504/512/546 ratio. The best test accuracy was achieved when using a learning rate of 0.01, batch size of 64, and 32 hidden LSTM units, as described in Table 4. Once hyperparameters are selected, we conducted a new training process using a tenfold evaluation approach, where 10 different models are trained and evaluated using ten fold splits (each time a model is trained on nine folds and tested on one fold). The achieved accuracy for the individual classes are as follows: neutrophil 89%, eosinophil 88%, basophil 57%, lymphocyte 93%, and monocyte 90%. The results are shown in Table 16, “Appendix 1”. The average accuracy of the five classes is 83%.

### Models comparison for WBC classification

Table 5 shows a summary of the results achieved by the different models, namely, W-Net, W-Net with SVM, AlexNet, VGGNet, ResNet, and RNN, using our dataset. The reported results are the average score of different accuracy metrics, accuracy, precision, recall, and F1-score. For W-Net, the accuracy, precision, recall, and F1-score are all 97%. For W-Net-SVM, they are 95%, 97%, 95% and 96% respectively. For Alexnet, they are 84%, 94%, 84% and 85% respectively. For VGGNet, they are 44%, 67%, 44% and 42% respectively. For ResNet, they are 51%, 60%, 51% and 43% respectively. For the RNN model, they are 83%, 86%, 85% and 85% respectively. The results show that W-Net outperforms other RNN- and CNN-based model’s architectures and an architecture with a small number of layers is also better than an architecture with many layers. The detailed results of tenfold cross validation for all experiments are in “Appendix 1”.

**Table 5:** The result of accuracy, precision, recall, F1-score on average and the number of layers for all experiments

Network	Acc. (%)	Prec. (%)	Rec. (%)	F1. (%)	# of layers
**W-Net**	**97**	**97**	**97**	**97**	**3**
W-Net-SVM	95	97	95	96	3
AlexNet	84	94	84	85	8
VGGNet	44	67	44	42	16
ResNet50	51	60	51	43	50
ResNet18	79	81	78	77	18
RNN	83	86	85	85	–

The parts in bold mean our model

### Further training with public data

The LISC public dataset [17] includes WBC images of size 720 × 576 × 3 that were collected from peripheral blood of eight normal people. The images are classified by a hematologist into five types of WBC: neutrophils, eosinophils, basophils, lymphocytes and monocytes. For pre-processing the public dataset, we cropped the WBC images (nucleus and cytoplasm regions) in the original images, and then re-sized the images to 128 × 128 × 3 for training. We used a total of 254 WBC images as our dataset: 56, 39, 55, 56 and 48 images for neutrophil, eosinophil, basophil, lymphocyte and monocyte, respectively. Using the LISC public data, this experiment shows the performance of W-Net when adopted for different datasets. Moreover, we show the performance of W-Net using transfer learning when a pre-trained W-Net is fine-tuned to classify WBCs from different dataset or used for different WBC-related tasks. To this end, we conducted two experiments as follows: ❶ W-Net architecture is used for building a WBC classifier trained using only the LISC public data, ❷ a pre-trained W-Net with softmax classifier from “W-Net performance” section is fine-tuned to classify WBCs from LISC public data. Except the training epochs, the training hyperparameters are set to be identical in both experiments. In the first experiment, W-Net-based model was trained from scratch using 4000 training epochs (254 × 4000/5 iterations) on the LISC public data. The training process was concluded after 10.33 h. In the second experiment, we establish a pre-trained W-Net-based model (trained on our dataset for 500 training epochs.) to be used on the LISC public data. The pre-trained W-Net-based model was fine-tuned for 4000 epochs (254 × 4000/5 iterations) on the public data. The training process was concluded after 10.83 h. Table 16 in “Appendix 1” shows the result of the first experiment where W-Net is used to classify WBCs from the LISC public data. The achieved results is an average accuracy of 91%. Table 17 in “Appendix 1” shows the result of the experiment. The average accuracy achieved using a pre-trained W-Net model is 96%.

In the results, the second experiment shows a better performance. This result shows that training a model in large-scale dataset (such as the one used for this study) can benefit other transfer learning tasks, where the model is fine-tuned to other dataset or performing other WBC-related tasks. We share our pre-trained model on GitHub [67] and believe that using the transfer learning property (transfer learning in the same domain) of deep learning models can help other researchers in the field.

## Design considerations for W-Net

Design choices for our deep learning architecture are described in this section. There are two challenging issues to consider in choosing a specific architecture in the large design space for WBC classification problem: One is how to figure out the data imbalance problem, and the other is to classify similar-looking images into the relatively small number of classes. In many datasets in real world, data imbalance is quite common and WBC images resembles way more each other compared to objects in traditional image classification problems. Also, the number of classes is quite limited compared with the traditional object identification problems such as ImageNet challenge. Therefore, it is necessary to take a different approach to the classification problem.

### Handling data imbalance: large batch and sampling

The results show that W-Net performs well despite the dataset’s imbalance, which is observed by the number of samples for each class. Even though the least-represented class in the dataset (basophil with 6% of the dataset) show the least accuracy of 95% in comparison to other classes, this accuracy is still higher than the results achieved by other methods, *e.g.,* CNN-based and RNN-based models, for the same class. This performance can be due to several reasons. For instance, the evaluation of all experiments follows a stratified k-folds cross-validation approach, which preserves the percentage of samples across all folds. Using this approach allows the sampling from all classes in different ratios in each fold, which dictates the inclusion of all classes in both the training and testing phases. When using a small batch size, *e.g.,* five samples as adopted during the training of W-Net, the error resulting from misclassifying one class, especially from underrepresented classes, highly impacts the average cost of the learning epoch and contributes in an effective learning process for these classes. In contrast, using a large batch size and considering a random sampling scheme for batching could result in minimizing the effect of misclassification of underrepresented classes since performing well on other classes could out-weigh the misclassification of small, if any at all, samples from classes with small ratios in the dataset.

Having different distributions of image samples per class is a hard part to classify WBC images. W-Net achieves an accuracy of 95% for identifying the basophil class which are represented with the least number of samples (377 samples and a ratio of 6% of the dataset). This result is remarkable knowing that all other CNN-based and RNN-based models achieved an accuracy below 56% and 57%, respectively, for the same class. The overall average accuracy of W-Net is 97%, which is the highest among other methods for WBC classification. Considering the results for this large-scale dataset, W-Net presents a state-of-the-art performance.

Furthermore, the result of W-Net with softmax classifier is 97%, the result of W-Net with SVM classifier is 95% and they seem similar. However, for the basophil class that has 6% distribution of our dataset, the accuracy of W-Net with SVM is only 87% and it is lower than 95% the result of softmax. The W-Net-SVM uses the hinge loss function, while W-Net uses the softmax cross-entropy loss function. The nature of optimization under these functions differs since the optimization using the hinge loss concludes when finding parameters that satisfy the classification with the predefined margin. However, using softmax cross-entropy loss keeps the optimization going beyond a specific margin pushing the decision boundaries further. This allows the model to maintain robust generalization capabilities, hence the better performance of W-Net over W-Net-SVM. AlexNet has many layers than our W-Net, however, the average accuracy is 84%, and especially the average accuracy of the basophil class that has 6% distribution of our dataset is 33%. This means the SVM classifier and the network of AlexNet are not appropriate to address the unbalanced dataset. As a result, we can claim that W-Net with softmax classifier is more effective than AlexNet and W-Net with SVM classifier in WBC image classification area.

### WBC dedicated architecture with shallow depth

In the tenfold cross-validation evaluation of W-Net, the minimum average accuracy is 91% (basophil, Fold-9) and maximum average accuracy is 100%. However, in the case of VGGNet and ResNet50 architectures which have more depth (considering the number of layers), the variance between the folds is from 0 to 100% resulting in 44% tenfold average accuracy, and from 0 to 100% resulting in 51% tenfold average accuracy, respectively. In a comparison between ResNet50 and ResNet18, since ResNet18 consists of a shallower layer than ResNet50, the overfitting problem seems to occur less. It leads that ResNet18 shows better performance with 79% on average than ResNet50. This means that very deep networks may not be the optimal choice for WBC image classification. Most of the state-of-the-art CNN-based models (e.g., AlexNet, VGGNet, and ResNet) use larger receptive fields, (e.g., 7 × 7 in case ResNet and 11 × 11 in the case of AlexNet), which seem to work better on larger images with larger objects (classes). However, handling the WBC classification task requires adopting smaller filters to bring attention to finer receptive fields that hold relevant features.

The results of this research show that architectures such as W-Net’s, which has five layers (three convolutional and two fully-connected.), can be sufficient and more effective in the WBC classification task in comparison to other deeper networks such as VGGNet, ResNet50 and ResNet18. In general, deep networks are known to perform well for the image classification, the VGGNet and ResNet with deep networks show good performance in ILSVRC. However, they did not show good performance in WBC image dataset. We claim that our dataset to be classified is different from the dataset aimed by those deep networks in two aspects: (1) the ILSVRC dataset has 1000 classes, but our WBC dataset has only five classes, and (2) The images of the ILSVRC dataset are very different from each other (For example, they are dog, bird, flower and food etc.), while our dataset has very high visual similarity.

To support this claim we conducted two simple experiments, ❶ the first experiment was to run W-Net on 200 classes (bird, ball and car etc.) of images from Tiny ImageNet dataset [68] and ❷ the second experiment was to run W-Net on five classes without visual similarity (fish, clothes, chair, car and teddy bear) from Tiny ImageNet dataset with the same (imbalance) distribution of our WBC dataset. In these two experiments, we only used different dataset with our WBC dataset, and used same network, parameters (learning rate and training epoch etc.) and tenfold cross-validation approach with our W-Net. In the first experiment, we used the dataset with 200 classes, and each class had 500 images. We used total 100,000 images. The result from the first experiment showed 100% accuracy for the 200th class, but 0% accuracy for the other 199 classes. The average accuracy was 0.5%, it showed that the model was not trained at all. In the second experiment, we used the dataset with 5 classes, and each class had 500, 333, 100, 433, 300 (making them have the same distribution with our dataset) images. We used total 1666 images. The result from the second experiment showed 34% accuracy for the third class (100 chair images), and 84%, 78%, 90% and 65% for other classes, respectively. The average accuracy was 79%, which was not as good as the results of W-Net using our dataset. Therefore, we claim that a simple network may be better to classify our WBC dataset with data distribution imbalance, small number of classes, and visual similarity.

### Why not RNN?

RNN-based models perform well in sequential data and show remarkable results in capturing temporal dependencies within the input data. There are different variations of RNN, and for our experiments we used LSTM models for their abilities to handle long-term dependencies (*e.g.,* 128 sequences in our application) and the vanishing gradient problem. The average achieved results when using one-layer LSTM model with 32 hidden units is 83%. This result is far from the results achieved by W-Net (97%).

However, it outperforms other CNN-based models such as VGGNet (44%) and ResNet50 (51%). Karol et al. [69] have also shown that RNN can encode independent scenes within an image instead of processing the entire image as a single input. Adopting sequential processing of white blood images via LSTM, enables the model to extract/adapt to patterns/changes in the scene to build a more robust model than following single-shot processing.

## Dataset sharing

Recent advances in big data have also led to advances in deep learning, accordingly having a good dataset has become important. In this section, we generate new WBC image samples using Generative Adversarial Networks (GAN) [18] then release them in public for education and research to help other researchers. GAN is a deep learning architecture for generating new artificial samples, it composes of two deep networks: ❶ the generator G, and ❷ the discriminator *D*. The *G* generates new samples from the domain, and the *D* classifies whether the samples are real or fake. The output of the *D* is used to update both the model weights of the *D* itself and the *G*. Accordingly, the performance of the *G* depends on how well the *D* performs. GAN can be expressed by: $\mathop {min }\limits_{G} \mathop {max }\limits_{D} V(D,G) = {\mathbb{E}}_{{x \sim p_{data} (x)}} [log D(x)] + {\mathbb{E}}_{{z \sim p_{z} (z)}} [log (1 - D(G(z)))]$, where *x* ~ *p*_*data*_(*x*) and *z* ~ *p*_*z*_(*z*) indicate the distribution of a real data and a fake data respectively, the *D* aims to maximize *logD*(*x*) and *G* aims to minimize *log*(1 − *D*(*G*(*z*))), to maximize the chance to recognize real images as real and generated images as fake. This expression defines GAN as a minimax game.

### Experimental settings

**Fig. 4:**
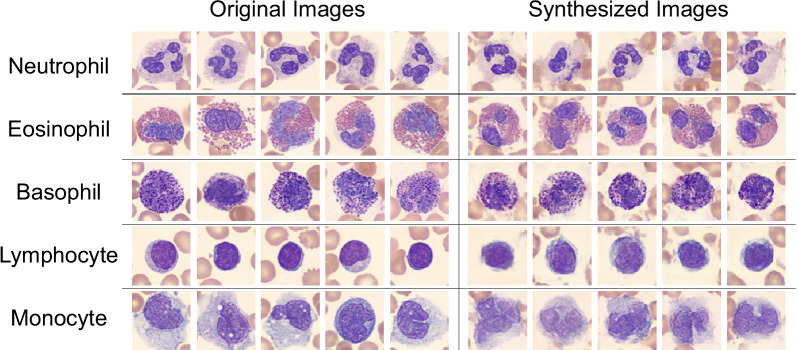
Left side: the original images of size of 128 × 128 × 3 for training DCGAN model. Right side: the synthesized images of size of 128 × 128 × 3 by trained DCGAN model. The first row is the neutrophil class, followed by the eosinophil, the basophil, the lymphocyte, and the monocyte classes

We use the same dataset (6562 WBC images of size of 128 × 128 × 3), similar experimental settings of previous experiments, and Deep Convolutional Generative Adversarial Network (DCGAN) [70] to train (*G* and *D*) models for generating images. For the network of *D*, six convolutional layers, one fully connected layer, LeakyReLU [71] activation, sigmoid activation and dropout are used. For the network of *G*, six convolutional layers, one fully connected layer, ReLU activation, sigmoid activation, dropout, and batch normalization [72] are used. The training hyperparameters are set as follows: alpha 0.2, momentum 0.9, batch size 1, learning rate 0.00001, dropout 0.6, and training epochs 10,000. The network of *G* and *D* have a total of 2,780,099 and 69,878,401 trainable parameters. It took 191.66, 120.13, 34.44, 158.33 and 91.66 h to train *G* and *D* models for five WBC classes, and it took an average of 18 min to generate 1000 images per each class. We generated 1000 plausible WBC images of size of 128 × 128 × 3 for each class (a total of 5000 images). Figure 4 shows the samples of both the original images (left side) for training DCGAN model and the generated images (right side) by trained DCGAN model. The first row of the Fig. 4 is the neutrophil class, followed by the eosinophil, the basophil, the lymphocyte, and the monocyte.

### Generated image quality

To see how similar images were generated from the original images, we verified the generated WBC images using ❶ baseline-W-Net, ❷ generative-W-Net (*i.e.,* W-Net trained on generated synthetic dataset), ❸ cosine similarity, and ❹ domain-expert experiment with a medical laboratory specialist. First, we experimented to classify the generated images using W-Net. Table 6 shows the confusion matrix for the results achieved for the classification of the generated WBC im-ages. The second column indicates true classes, the second row indicates predicted classes, and the images are well-classified with 100% accuracy by W-Net. Second, we trained W-Net model using the 5000 generated synthetic images. For the training, we follow the same experimental settings of creating the baseline-W-Net. Then, we evaluated the generative-W-Net for classifying the 6562 real WBC images. Table 7 shows the confusion matrix for the results achieved for the classification of real WBC images using generative-W-Net. The images are classified with an accuracy of 95%, precision of 93%, recall of 95%, and F1-score of 94%. Third, we measure the similarity between the original images and the generated images using cosine similarity. We first measure the cosine similarity between the original images and the original images for each class (*e.g.,* 377 vs. 377 for the basophil class), then we measure the cosine similarity between the original images and the 1000 generated WBC images for each class (*e.g.,* 377 vs. 1000 for the basophil class) and then we compare them. Table 8 shows the difference in the cosine similarity between the original images and generated images. It was 4% for the neutrophil, 3% for the eosinophil, 7% for the basophil, 6% for the lymphocyte, and 6% for the monocyte with average 5% for five classes. Fourth, we conducted a domain-expert experiment on how well a medical laboratory specialist could classify the generated WBC images. The dataset used in this experiment consists of 10 random original images and 10 random generated images for each class, *i.e.,* a total of 100 images. Without informing the medical laboratory specialist of the source of the WBC images in the dataset, we asked for the classification of provided images. Table 9 shows the confusion matrix for this experiment. The results show that the specialist well-classified the given WBC samples with an accuracy of 95%. Among the five misclassified images, there are three original images and only two generated images. The results of all verification methods for the generated images show that the generated images are similar to the original images. We released the generated (labeled) WBC images on GitHub [67] for the education and research purposes.

**Table 6:** The confusion matrix for classification experiment result with generated WBC images using W-Net model

	Predicted classes				
	NE.	EO.	BA.	LY.	MO.
True classes					
NE.	1000	0	0	0	0
EO.	0	1000	0	0	0
BA.	0	0	1000	0	0
LY.	0	0	0	1000	0
MO.	0	0	0	0	1000

The images were well-classified with 100% accuracy

**Table 7:** The confusion matrix for classification experiment result with real WBC images using the fake W-Net model

	Predicted classes				
	NE.	EO.	BA.	LY.	MO.
True classes					
NE.	1979	1	19	5	2
EO.	11	1273	19	7	0
BA.	7	3	355	10	2
LY.	8	2	59	1572	35
MO.	8	0	9	77	1099

The images were classified with 95% accuracy

**Table 8:** The difference in the cosine similarity between the original images and generated images

	NE.	EO.	BA.	LY.	MO.	Aver.
Cos. Sim.	4%	3%	7%	6%	6%	5%

**Table 9:** The confusion matrix for the user experiment result with the medical laboratory technologist

	Predicted classes				
	NE.	EO.	BA.	LY.	MO.
True classes					
NE.	19	0	0	1	0
EO.	0	19	0	0	1
BA.	0	0	20	0	0
LY.	1	0	0	19	0
MO.	2	0	0	0	18

The technologist classified the generated WBC images with 95% accuracy

## Conclusion

Analysis of WBC images is essential for diagnosing leukemia. Although there are several methods for detecting and counting WBCs from microscopic images of a blood smear, the classification of the five types of WBCs is still a challenge in real-life applications, which we addressed in this work. The rapid growth in the area of computer vision and machine/deep learning have provided feasible solutions to classification tasks in many domains. This work proposed W-Net, a CNN-based architecture with a small number of layers, to accurately classify the five WBC types. We evaluated W-Net on a real-world large-scale dataset and addressed several challenges such as the transfer learning property and the class imbalance. W-Net achieved an average classification accuracy of 97%. Moreover, we compared the result of W-Net with W-Net with SVM, AlexNet, VGGNet, ResNet and RNN architectures to show the superiority of W-Net which consists of three layers over other architecture. We synthesized a dataset of new WBC image samples using DCGAN, which we released to the public for education and research purposes.

Even though our W-Net model provides good performance with an average classification accuracy of 97%, it still remains an error of 3%. In the future work, we will conduct the dataset augmentation using our generative model based on DCGAN, to address the dataset imbalance. Then, we will carry out additional experiments to further increase the accuracy performance of the classification model with the balanced dataset.

## Data Availability

Our pre-trained model and the generated WBC images is available for scientific and education purposes on on GitHub [67] (https://bit.ly/3jfB7yA, https://bit.ly/3pM3Ptf).

## Appendix 1: The detailed results for all experiments

In this section, we show the detailed results of tenfold cross validation for W-Net (Table 10), W-Net-SVM (Table 11), AlexNet (Table 12), VGGNet (Table 13), ResNet (Tables 14, 15), RNN (Table 16) and further training (Tables 17, 18).

**Table 10:** The result of tenfold cross-validation of W-Net for classification accuracy

	NE. (%)	EO. (%)	BA. (%)	LY. (%)	MO. (%)
Fold-0	100	95	92	99	96
Fold-1	98	99	94	100	100
Fold-2	96	93	100	95	98
Fold-3	97	99	100	95	96
Fold-4	100	100	97	98	97
Fold-5	100	98	94	97	98
Fold-6	100	98	94	97	91
Fold-7	95	98	94	98	96
Fold-8	100	93	94	97	99
Fold-9	98	100	91	95	97
Avr. Acc.	98	97	95	97	97

The average accuracy for five classes is 97%

**Table 11:** The result of tenfold cross-validation of W-Net-SVM for classification accuracy

	NE. (%)	EO. (%)	BA. (%)	LY. (%)	MO. (%)
Fold-0	100	96	78	100	99
Fold-1	100	94	89	100	96
Fold-2	85	97	97	97	97
Fold-3	97	94	89	97	91
Fold-4	98	99	86	99	98
Fold-5	100	99	78	96	100
Fold-6	100	98	89	98	94
Fold-7	96	97	89	100	92
Fold-8	100	95	86	98	96
Fold-9	99	98	91	97	97
Avr. Acc.	98	97	87	98	96

The Aver. Acc. for five classes is 95%

**Table 12:** The result of tenfold cross-validation of AlexNet for classification accuracy

	NE. (%)	EO. (%)	BA. (%)	LY. (%)	MO. (%)
Fold-0	98	96	13	98	100
Fold-1	97	98	45	98	98
Fold-2	88	98	58	95	99
Fold-3	96	100	18	90	97
Fold-4	98	100	18	94	99
Fold-5	100	99	29	90	100
Fold-6	99	98	47	92	97
Fold-7	92	98	27	99	98
Fold-8	99	98	35	92	100
Fold-9	100	99	41	86	99
Avr. Acc.	97	99	33	93	99

The Aver. Acc. for five classes is 84%

**Table 13:** The result of tenfold cross-validation of VGGNet for classification accuracy

	NE. (%)	EO. (%)	BA. (%)	LY. (%)	MO. (%)
Fold-0	100	2	21	0	32
Fold-1	100	0	0	0	75
Fold-2	100	3	31	0	57
Fold-3	100	87	47	16	12
Fold-4	100	84	81	4	74
Fold-5	100	33	0	20	89
Fold-6	100	0	7	40	68
Fold-7	100	44	2	1	12
Fold-8	100	62	16	0	51
Fold-9	100	64	21	8	57
Avr. Acc.	100	38	23	9	53

The Aver. Acc. for five classes is 44%

**Table 14:** The result of ResNet50 for classification using tenfold cross-validation

	NE. (%)	EO. (%)	BA. (%)	LY. (%)	MO. (%)
Fold-0	100	0	0	49	1
Fold-1	0	16	26	94	50
Fold-2	100	90	94	5	100
Fold-3	99	95	100	81	100
Fold-4	0	1	78	67	1
Fold-5	0	23	5	100	24
Fold-6	0	98	86	0	100
Fold-7	100	1	10	54	1
Fold-8	100	95	100	33	23
Fold-9	0	87	56	0	100
Avr. Acc.	50	51	56	48	50

The average accuracy for five classes is 51%

**Table 15:** The result of ResNet18 for classification using tenfold cross-validation

	NE. (%)	EO. (%)	BA. (%)	LY. (%)	MO. (%)
Fold-0	96	99	53	61	76
Fold-1	83	94	97	97	89
Fold-2	54	21	86	73	91
Fold-3	89	92	70	68	84
Fold-4	74	92	100	85	82
Fold-5	86	82	56	74	69
Fold-6	84	93	100	88	62
Fold-7	70	91	90	99	93
Fold-8	61	81	61	73	42
Fold-9	97	86	100	83	71
Avr. Acc.	79	83	81	80	75

The average accuracy for five classes is 79%

**Table 16:** Tenfold evaluation of LSTM (RNN) model

	NE. (%)	EO. (%)	BA. (%)	LY. (%)	MO. (%)
Fold-0	92	88	55	94	90
Fold-1	86	81	55	88	92
Fold-2	86	85	55	90	87
Fold-3	90	93	71	94	92
Fold-4	94	93	73	96	91
Fold-5	92	88	50	94	91
Fold-6	89	93	65	93	86
Fold-7	88	92	56	94	89
Fold-8	81	84	40	94	92
Fold-9	89	84	51	95	90
Avr. Acc.	89	88	57	93	90

The average accuracy for five classes is 83%

**Table 17:** The result of the first model trained using LISC public data from scratch

	NE. (%)	EO. (%)	BA. (%)	LY. (%)	MO. (%)
Fold-0	33	100	50	100	60
Fold-1	83	100	83	100	100
Fold-2	100	100	100	100	20
Fold-3	100	100	100	100	80
Fold-4	83	100	100	83	100
Fold-5	83	100	100	100	100
Fold-6	100	100	100	100	100
Fold-7	80	100	100	80	100
Fold-8	80	100	80	100	100
Fold-9	100	100	80	100	100
Avr. Acc.	84	100	89	96	86

The average accuracy for five classes is 91%

**Table 18:** The result of the second model was initially trained using our dataset which is our W-Net model and then further trained using LISC public data

	NE. (%)	EO. (%)	BA. (%)	LY. (%)	MO. (%)
Fold-0	100	100	100	100	80
Fold-1	100	100	100	100	100
Fold-2	100	100	100	100	20
Fold-3	100	100	100	100	100
Fold-4	100	100	100	100	80
Fold-5	100	100	100	83	100
Fold-6	100	100	100	100	100
Fold-7	100	75	100	100	100
Fold-8	100	100	80	100	100
Fold-9	100	100	80	100	100
Avr. Acc.	100	98	96	98	88

The average accuracy for five classes is 96%
